# The comparative ethics of artificial-intelligence methods for military applications

**DOI:** 10.3389/fdata.2022.991759

**Published:** 2022-09-12

**Authors:** Neil C. Rowe

**Affiliations:** Department of Computer Science, U.S. Naval Postgraduate School, Monterey, CA, United States

**Keywords:** artificial intelligence, ethics, algorithms, military, lethal autonomous systems, explanation, transparency

## Abstract

Concerns about the ethics of the use of artificial intelligence by militaries have insufficiently addressed the differences between the methods (algorithms) that such software provides. These methods are discussed and key differences are identified that affect their ethical military use, most notably for lethal autonomous systems. Possible mitigations of ethical problems are discussed such as sharing decision-making with humans, better testing of the software, providing explanations of what is being done, looking for biases, and putting explicit ethics into the software. The best mitigation in many cases is explaining reasoning and calculations to aid transparency.

## Introduction

Artificial-intelligence (AI) software is increasingly proposed to replace humans in military technology and military planning involving potential lethal force. Military conflict is dangerous, and there is much incentive to automate its actors. For instance, an automated gun turret from South Korea that uses simple AI is internationally popular (Parkin, 2015) although its ethical principles have not been carefully evaluated. The most obvious ethical issues with military AI occur with targeting, and other issues arise in the planning of operations and logistics support. However, building AI systems to make potentially lethal judgments is difficult, and current AI methods are still less accurate than humans for many tasks (Emery, 2021). Using them to apply lethal force can be unethical, just as using an imprecise weapon like a shotgun in military conflict today. Furthermore, a major justification for the use of lethal force in the laws of armed conflict is self-defense, something less relevant to software and robots since they can be cheaply remanufactured, although limited self-defense could still be appropriate for them to preserve their capabilities during an ongoing conflict. So it is important to assess how each AI method works to see how well its contribution to lethal force can be justified, and the methods differ considerably in their accuracy and explainability, and hence their possible justifiability.

Not all ethical problems of AI systems can be blamed on the software, as problems can be due to errors in input data, misconfiguration of systems, or deliberate sabotage. Furthermore, many software problems of AI systems cannot be blamed on AI, since AI depends on man-machine interfaces, databases, and networking that can also be faulty. Also, successful development of AI, like that of other software, depends on familiarity with the context in which it will be used, and AI developers rarely have experience in warfare that they can use in developing military AI systems. We do not consider those problems here as we wish to focus exclusively on problems of AI methods in this short article. Note that AI methods are not necessarily less ethical than those of humans, since such methods can exceed human capabilities in speed, accuracy, and reliability when designed, debugged, and tested well, and this could enable them make ethical decisions more accurately than humans in challenging situations.

## Artificial-intelligence methods

Artificial intelligence is generally considered to be methods for creating intelligent behavior with software, not necessarily human methods. It is a form of automation, and raises some of the same issues as other kinds of automation, plus a few new ones because of its focus on information rather than machinery. Most ethical theories ascribe blame for unethical actions of algorithms (methods) to their creators and deployers; (Tsamado et al., 2021) identifies a wide range of possible ethical issues with algorithms. AI algorithms fall into several categories as described in (Rowe, 2022):

- Logical inference methods that reach yes/no conclusions assuming a set of starting facts. This includes rule-based systems with if-then rules, decision trees, and reasoning by analogy.
- Uncertain inference methods that reach conclusions with an associated degree of certainty, assuming a set of starting assertions with probabilities. This includes most artificial neural networks, Bayesian reasoning, and case-based nearest-neighbors reasoning.
- Planning and search methods that find good sequences or plans to solve problems using logical reasoning. This includes heuristic depth-first search, heuristic breadth-first search, and hierarchical planning.
- Planning and search methods that find good sequences or plans with an associated degree of estimated quality or certainty. This includes A^*^ search, game search, and recurrent neural networks.
- Machine learning (the usual name for learning methods in the AI field) and other optimization of logical inference methods from examples of desired behavior. This includes set-covering methods, decision trees, and support-vector machines.
- Machine learning and optimization of uncertain inference methods from examples of desired behavior. This includes backpropagation and other optimizations of artificial neural networks.
- Machine learning and optimization of planning and search methods from examples from desired behavior. This includes reinforcement learning and optimization of recurrent neural networks.
- Machine learning and optimization of AI algorithms without examples of desired behavior (“unsupervised learning”). This includes clustering, principal-components analysis, latent semantic analysis, generative adversarial networks, and evolutionary algorithms.
- Reasoning methods that imitate those of humans or groups of humans. This includes implementations of a wide range of psychological theories.

Like other software, AI software can be assessed by several kinds of metrics:

- Accuracy of its logical reasoning. This is usually applied to classification tasks, and two classic metrics are correctness in what it classifies (precision), and completeness in what it classifies (recall).
- Accuracy of its numeric inferences: Average closeness to the correct answers using some error metric.
- Speed of its reasoning.
- Storage space required for its reasoning.
- Robustness in handling errors in its input
- Ability to explain its results and how it got them.
- Similarity of the results to human reasoning.

All these metrics bear on ethics. Most are predominantly quantitative. Thus they can be a basis for utilitarian ethics, or a basis for deontological ethics if we assign labels to ranges of numbers and refer to labels. However, the last two metrics above are more qualitative and need different assessment techniques.

## Possible improvements to the ethics of AI algorithms

Ethical issues with AI software can be mitigated in several ways. The major ones are considered here.

### Putting humans in the decision-making loop

A key worry with AI software is whether it can be trusted to think and act substantially as humans do, on the assumption that humans are generally more ethical than machines since humans have higher-level goals. Subissues are whether AI systems can know everything that humans do to make decisions and whether they will reason similarly to humans with the same information. When these are concerns with military decisions, particularly those about lethal force, humans should be involved (“in the loop”); for instance, humans may know additional reasons that the AI does not as to why civilians are more likely to appear in a combat zone. Teams of carefully selected humans could also provide more diversity of points of view than AI could. AI could then serve an advisory role, recommending courses of action that could be overruled by human superiors. Many battle-management systems using AI are like this today.

However, such “hybrid” man-machine systems are not necessarily more ethical than machines alone (Cummings, 2021). Human personnel may not have access to the potentially huge amount of data and options that software might have, and so might make worse decisions than the software. Humans also have biases which can cause them to make bad decisions. These include well-known flaws in reasoning (Kahneman et al., 1982) such as a tendency to predict what they have seen before, something dangerous in military conflict where deception is often involved. Humans can also be influenced by propaganda, and can have deliberate unethical intentions. So putting humans in a decision loop will not necessarily ensure more ethical behavior.

### Testing of AI systems

Some ethical problems with AI systems can be mitigated with proper testing. Software is complex and can easily contain harmful mistakes or flaws, that might cause lethal force to be used when the software designers did not intend so. Work on critical software has developed many testing methods to find bugs and flaws. Since most systems have too many possible inputs to test them all, sampling methods are essential though not guaranteed to find all bugs and flaws. A popular technique is “fuzzing” which tries small variants of tested input patterns to see if unusual effects occur.

Still, flaws in software are found all the time after it is released, and some of them can cause harm. Flaws are not always quickly reported publicly or quickly fixed after discovery (Lidestri and Rowe, 2022). Inadequate testing is common since incentives are weak for vendors to thoroughly debug before releasing software, and some vendors wait for users to find most bugs for them. It is difficult for users to recognize many bugs by themselves; many safety-related features in software are invoked rarely, so users cannot tell if they work properly. Nonetheless, many software vendors are conscientious, and voluntarily search for bugs.

Testing of AI machine-learning methods such as unsupervised learning that make random choices is particularly difficult. Such methods may give different answers when trained at different times on the same data, much less on different data. A solution is “cross-validation” where systems are trained to build models on random subsets of the data, and a consensus of the trained models taken as the result.

### Explanation facilities: Inference

Lack of flaws alone is not enough to claim that AI software has acted ethically since its design may have other weaknesses. This is especially important with targeting, which can require careful judgement. Explanation capabilities can show how the software made its decisions, as a form of “transparency.” Explanations also help debugging and provide legal justifications of AI (Atkinson et al., 2020). For software that does logical reasoning, an explanation can show the input data and the sequence of logical inferences made with it. For instance, if software identified a vehicle as hostile, an automatically generated explanation can show which features of the vehicle were relevant and what inferences supported the conclusion that it was hostile. Many AI systems that do logical reasoning make only a few logical inferences for a conclusion, and a trace of those will not overwhelm humans. Even better, we can allow users to ask “why” questions for particular conclusions made about the data that will identify just the data and inferences used. For instance, a system may conclude a vehicle is hostile if it has markings particular to an adversary and is in a location known to be controlled by an adversary.

For AI that does numerical calculations, explanation of decisions is harder. Typically such systems check whether the result of a calculation is over a threshold. The calculation is usually far too complex to explain to humans, especially with artificial neural networks. This raises problems for ethics because incomprehensibility prevents easy justification of the method. Some work on neural networks has tried to explain conclusions better; for instance, we can measure the impact of each factor or network feature on the complex mathematical function. However, this may not help much because often the correlations between factors matter more than the factors individually, and there are many possible correlations. To address this, some approaches try to identify larger parts of a neural network that have more impact on a conclusion, called areas of highest “salience” (Jacobson et al., 2018). However, this may not provide a good explanation either.

Explanations for military data could require revealing sensitive or classified data, such as data obtained by secret equipment. A less revelatory method may be to provide unclassified “precedents” for the case being explained. If they predominantly demonstrate the same conclusion as the case, the precedents and their reasoning can be presented. A challenge of this is defining similarity between cases: Some differences should be given higher weights based on machine learning from examples.

### Explanation facilities: Planning

AI can also be used to plan military operations. If unethical operations such as deliberate targeting of civilians are planned by AI, the result will be unethical regardless of the accuracy of the targeting software. Unfortunately, many planning systems are focused only on sensors, weapons, and logistics.

The ethics of plans generated by AI methods can be improved by calculating and displaying their ethical factors explicitly, such as possible civilian casualties of a plan or the risk of exerting disproportionate force. As with inference, explanations of plans can enable scrutiny and easier detection of ethical issues, up to some limits of complexity (Ananny and Crawford, 2016). Helpful explanations for logically-generated plans can reference preconditions, postconditions, and priorities on actions. “Why” questions about actions can be answered by relative costs and benefits, or by preconditions in a hierarchy of goals. But complex numerically-based plans can be hard to justify. Explaining targeting may require not only analysis of the costs and benefits of each target but the resources available and the logistics of getting them to the targets, and the tradeoffs can be complex. A simpler numeric model that can explain a similar plan can help, as for instance a Bayesian conditional-probability model rather than a deep neural network.

### Looking for biases

AI systems can perpetuate unfair biases, particularly when they are developed using machine-learning methods on complex data. For instance, an AI system may be trained on U.S. data in which friendly forces were tall, and thus be more inclined to identify short people as combatants; or it may be trained on indicators of aggression seen in one part of the world, like maneuvers along a frontier, that may not occur elsewhere. Bias is particularly troublesome for AI systems because the bias may be deeply hidden in a large amount of data and no one may be aware of it. Some of these situations exemplify a well-known problem of statistical sampling of getting a representative sample of input. If important types are underrepresented in the raw data, data can be duplicated, or frequent types can be reduced in number (subsampled). Better transparency of systems by explanation tools can also help the analyses of their biases.

### Automated ethical reasoning

Another way to improve the ethical behavior of AI software is to design the AI itself to use explicit ethical principles or criteria such as those of (Galliott, 2021). For instance, the principle of avoiding threats to civilians can be modeled by building a separate neural network that calculates the expected number of civilians to be harmed near a target based on intelligence data (Devitt, 2021) provides a start at a set of implementable principles. People seem to understand deontological ethics more easily than utilitarian ethics, so the principles will be easier to understand and justify if expressed as if-then rules. They will require setting thresholds on probabilities and other quantities, so designers must be prepared to argue why a 0.6 probability of killing a civilian is acceptable. Nonetheless, automated ethical principles could be better than human decision-making since they can avoid emotional responses to particular nationalities, ethnicities, political groups, or religions and thus could judge threats more objectively.

## Recommendations

This article has discussed several ways to improve the ethics of AI systems, but the most important is transparency of their operations in the form of explanations of what they are doing. Thus, ethical AI methods should be simple to explain and easy to justify. Numerical AI methods like artificial neural networks are more likely to be problematic because the complexity of their calculations makes them difficult to explain. Methods requiring long logical reasoning chains of if-then rules cause similar problems. Numerical methods also often require thresholds for action (like the speed of a missile to entail a response) which can be difficult to justify, and this is especially a problem for decision-tree and support-vector methods. Unsupervised machine-learning methods are also problematic because they are hard to control.

These issues mean it is also important to reveal the algorithms and key details of AI software used for military applications so that potential ethical risks can be identified. Some ethical issues can also be monitored automatically from within AI software, such as by estimating the casualties of a course of action and using that in recommending decisions.

## Data availability statement

The original contributions presented in the study are included in the article/supplementary material, further inquiries can be directed to the corresponding author.

## Author contributions

The author confirms being the sole contributor of this work and has approved it for publication.

## Funding

This work was supported in part by the U.S. National Artificial Intelligence Institute, the U.S. Department of Veteran's Health Affairs, and the U.S. Department of Defense Office of Research and Development.

## Conflict of interest

The author declares that the research was conducted in the absence of any commercial or financial relationships that could be construed as a potential conflict of interest.

## Publisher's note

All claims expressed in this article are solely those of the authors and do not necessarily represent those of their affiliated organizations, or those of the publisher, the editors and the reviewers. Any product that may be evaluated in this article, or claim that may be made by its manufacturer, is not guaranteed or endorsed by the publisher.

## Author disclaimer

The views expressed are those of the author and do not represent the U.S. Government.
